# Oncology Treatment in India: A Narrative Exploration of Patient Engagement and Care Strategies

**DOI:** 10.7759/cureus.87329

**Published:** 2025-07-05

**Authors:** Shamsuzzaman Ansari, Geetika Patel, Anushka Malaviya, Vaishnavee Dehankar, Rajeswari BS

**Affiliations:** 1 Pharmaceutical Management, Institute of Health Management Research, Bangalore, IND; 2 Hospital Management, Institute of Health Management Research, Bangalore, IND; 3 Public Health, Institute of Health Management Research, Bangalore, IND

**Keywords:** community-initiatives, family involvement, patient centered care, patient engagement, patient involvement, shared decision making

## Abstract

The growing burden of cancer poses a significant public health challenge, with late-stage diagnoses leading to severe health complications. As a life-altering disease, cancer not only impacts individuals but also affects their families, communities, and the nation as a whole. Effective patient engagement is crucial for improving health outcomes, and while India has made considerable progress in this area, several challenges persist. This narrative review aims to explore patient engagement and care strategies implemented by oncology healthcare providers across India. In order to do this, a comprehensive search was conducted across PubMed, ScienceDirect, and Google, using keywords such as patient engagement, patient engagement in oncology, patient engagement by cancer care hospitals, community engagement, community outreach, and patient-centered care by oncology service providers. Websites of key oncology service providers were also explored to identify the patient engagement and care strategies implemented by these service providers. The review specifically examines research from India to ensure relevance to the country’s healthcare landscape. These strategies include patient education and empowerment, technology-driven innovations, community-based initiatives, patient-centered communication, health gamification and incentive programs, and caregiver and family involvement. Findings indicate that enhancing community participation, expanding financial support programs, integrating artificial intelligence (AI)-powered digital health solutions, and increasing telemedicine accessibility can significantly improve patient involvement and health outcomes. The review found numerous barriers that hinder widespread adoption, including disparities in healthcare access, low health literacy, lack of awareness and motivation, technological challenges, increasing out-of-pocket expenses, an insufficient medical workforce, and limited cancer research. Systematically addressing these challenges is essential for establishing an inclusive, accessible, and patient-centered oncology care system in India.

## Introduction and background

Patient engagement (PE) has gained substantial momentum in recent years, fueled by shifting patient and caregiver expectations, technological advancements, and an increasing acknowledgment of its role in enhancing healthcare quality and patient outcomes. Patient engagement refers to the active participation of individuals in managing their health. This involves taking part in the decision-making process, managing your healthcare needs, and collaborating with healthcare facilities and providers. It is an important aspect of value-based care, which aims to improve quality care along with low costs [1]. Healthcare industry stakeholders, primarily treatment and solution providers, consider patient engagement a critical strategy [2]. Cancer care is no exception. Modern cancer patients seek more than just treatment; they expect a comprehensive care program that actively involves them in decision-making, educates them about their treatment options, keeps them informed about their progress, and ensures they are treated with respect and dignity [3]. Hence, patient involvement is increasingly significant in cancer care, and nurses play a vital role in encouraging those with cancer to take an active part in their treatment and well-being. [4]. For effective collaboration, physicians and cancer program staff must be receptive and willing to dedicate additional time to patients and their caregivers, ensuring their active involvement in the care process.

Cancer remains a major public health, societal, and economic burden in the 21st century, contributing to approximately one in six deaths (16.8%) worldwide, nearly one in four (22.8%) deaths from non-communicable diseases (NCDs) [5]. In 2020, the most frequently diagnosed cancers included breast cancer (2.26 million cases), lung cancer (2.21 million cases), colorectal cancer (1.93 million cases), and prostate cancer (1.41 million cases). That same year, the primary cause of cancer-related mortality was lung cancer (1.8 million cases), followed by colorectal cancer (916,000 deaths) [6].

India is undergoing an epidemiological transition marked by a rising prevalence of non-communicable diseases, with cancer emerging as a critical health challenge. India ranks third in cancer incidence globally, following China and the United States (USA) [7]. As per the report by the Indian Council of Medical Research (ICMR), an estimated 2.5 million individuals in India (roughly twice the population of Hawaii) are currently living with cancer, with nearly 700,000 new cases being added annually [8]. Additionally, it is projected that approximately one in nine Indians will be diagnosed with the cancer at some point in their lifetime [9]. Among males, lung cancer ranks the highest, while breast cancer remains the most common among females. Among age groups 0 to 14, lymphoid leukemia accounts for 29.2% of cases in boys and 24.2% in girls. By the end of 2025, India’s cancer burden is anticipated to reach 29.8 million disability-adjusted life years (DALYs), with the northern and northeastern regions experiencing the greatest impact. Contributing to the highest prevalence and mortality rates are challenges such as insufficient healthcare infrastructure, delays in diagnosis, and limited access to quality oncology care, especially in rural areas.

The rising cancer burden is compounded by the financial strain it places on work-life balance, decreased productivity, and affects relationships. The patient seeks emotional support not just from their family, but also from healthcare providers and the community. Each of them is essential in improving patients’ health outcomes [9, 10]. Addressing these challenges requires a holistic approach. Earlier in India, the responsibility of making treatment decisions primarily rested with doctors. But now decision making towards their ailment is considered a patient's right [11]. As the system's complexity (for example, multiple appointments, insurance claims, decisions towards treatment options) increases yearly, healthcare systems are now making efforts to involve patients and their caregivers for better health outcomes and experiences, and innovating their strategies to support patients. Involving patients with doctors and nurses to take active participation in their treatment journey keeps patients motivated and enthusiastic towards their health, and this also maintains transparency with the patients.

Research has shown that actively involved patients experience better health outcomes, while low engagement is linked to an increased risk of adverse events [11]. Increased awareness about diseases and available treatment options plays a crucial role in empowering both patients and healthcare providers. When patients are well-informed, it enables shared decision-making, fosters trust, and leads to more personalized care, ultimately contributing to better health outcomes. Strategies like personalized treatment plans, technology-driven solutions, and involving patients and their families in the journey help in bridging the gaps in their cancer care delivery. Over the past few years, India has evolved significantly, driven by innovation and a commitment to addressing these needs.

The Indian oncology market was projected to reach approximately USD 3.02 billion by 2024, driven by rising awareness, early detection, and widespread adoption of diagnostic technologies [12]. As per market research firm Technavio, the Indian oncology market is projected to grow by USD 2.02 billion, with a compound annual growth rate (CAGR) of 19.8% from 2024 to 2029. This market includes diagnostics, treatment, and therapeutics for various chronic diseases, with a primary focus on cancer [13]. India is advancing in terms of healthcare facilities, allowing cancer patients with medical care along with progress in technology, which positions India as one of the leading players for oncology treatment [14]. Leading hospitals offering cancer treatment in India include Apollo, HealthCare Global Enterprises Ltd. (HCG), Fortis, Max Healthcare, The Madras Institute of Orthopaedics and Traumatology (MIOT) International, and Artemis Hospitals, among others [15].

The primary aim of this review is to examine the diverse patient engagement strategies implemented by leading hospitals across India to enhance cancer care delivery. The article also explores innovative approaches designed to improve patient involvement, treatment adherence, and the overall healthcare experience. Furthermore, it identifies persistent barriers and challenges in cancer care, such as issues related to accessibility, affordability, and infrastructure, and provides insights into potential solutions for strengthening oncology services on a national scale. Given the narrative nature of this review, the qualitative approach has been adopted to map the current practices and trends. The article includes an overview of key patient engagement strategies in oncology care in India, examples and illustrations from leading oncology hospitals, identification and discussion of key challenges in patient engagement, and finally suggestions for improving patient-centric cancer care models.

## Review

Methodology

This article is a narrative review article, exploring patient engagement strategies implemented by oncology service providers and hospitals in India. While it includes research papers dating back to 2010, the primary emphasis is on studies and articles that reflect the changes in the healthcare landscape following the pandemic. A comprehensive search was conducted across PubMed, ScienceDirect, and Google, using keywords such as patient engagement, patient engagement in oncology, patient engagement by cancer care hospitals, community engagement, community outreach, and patient-centered care by oncology service providers. The review includes empirical research, commentaries, editorials, and qualitative studies. In the absence of sufficient peer-reviewed academic literature specific to patient engagement strategies in Indian oncology settings, secondary sources for data collection such as websites of prominent oncology hospitals to extract information on current patient engagement initiatives, blog posts, news articles, and industry reports were also referred to gain a broader understanding of evolving practices and contextual challenges. This approach allowed for to synthesis of diverse perspectives and practical implementations, providing a more grounded understanding of real-world practices. All above-mentioned sources of information were selected based on their focus on oncology services specifically in India, whereas studies with a non-oncology or global perspective without any relevance to the Indian context were excluded.

Key patient engagement strategies in cancer care

Patient engagement includes delivering information by healthcare systems and acknowledging the preferences of individuals. This allows patients to take informed decisions and proactive steps to improve health outcomes. It is a marker of the patient’s ability to showcase their resilience towards their treatment and be an active participant instead of a mere observer in their healthcare journey [16]. Innovations in technology have broadened access to information, allowing patients to have a better understanding of the disease and treatments available. Globally active engagement of patients has become a key focus, ensuring adherence to treatment and compliance with treatment protocols. An essential component of patient engagement is the doctor-patient relationship, which builds a foundation for effective communication and collaborative care. Patients must trust their healthcare providers to achieve a desirable treatment outcome, leading to satisfaction and adherence to treatment. When patients trust their healthcare providers, they feel more comfortable sharing their concerns, asking questions, reporting adverse events and side effects, and discussing their treatment options [17]. Open communication enables healthcare professionals to gain a deeper understanding of a patient’s expectations, needs, and preferences, paving the way for personalized and patient-centered care.

Involving patients in their cancer care is vital and should not be ignored. Patient engagement is vital in oncology care as the treatment is complex and demands collaborative decision-making between patients and healthcare providers [18]. Inadequate, comprehensive, and coordinated follow-up care for survivors can lead to challenges in their transition post-treatment, increasing the risk of cancer recurrence [19-20]. Indian healthcare providers implement several key patient engagement strategies, including the following:

Patient Education and Empowerment

As cancer care is shifting from acute to chronic management, it is essential to equip patients with knowledge, confidence, and resources, enabling them to show active participation in their care [21]. This involves perceived care quality, control over treatment, and strong communication with healthcare providers and caregivers [22]. When patients understand their diagnosis, treatment options, and medical instructions, they can make informed decisions that align with their values and preferences. This also helps them to communicate with the healthcare team, manage side effects, and access necessary support services [23]. Recognizing this, initiatives like The Lung Connect (Buddy Program) and Knowledge, Empowerment, Virtual Access, and Treatment (KEVAT) by Tata Memorial Hospital, Mumbai [24-25], as well as the Apollo Cancer Centre (ACC) support groups by Apollo Hospitals [26], have undertaken structured efforts to educate and support patients. These programs connect cancer survivors with newly diagnosed patients, offering emotional support, sharing practical experiences, fostering resilience, and encouraging active participation in the cancer care journey.

Technology and Digital Innovations

As India advances as a developing nation and moves towards becoming a technology-driven economy, the role of technology in healthcare has become indispensable, touching the lives of millions. Digital health can be defined as the use and application of digital tools to improve healthcare services and delivery. These healthcare technologies can be utilized throughout the patient’s journey, starting from diagnosis and treatment to follow-up care. For example, “ASyMS is an advanced symptom management system utilizing mobile phone technology for patients to report cancer chemotherapy-related symptoms [27]. Kaiku Health is an electronic patient-reported outcome web-based platform developed to monitor symptoms and quality of life in cancer patients. It offers real-time and current cancer data as well as support for patients and healthcare professionals [28]. OncoBuddy, a health app that provides patient patient-centric ecosystem that offers holistic support to cancer patients. It connects patients, provides awareness, and empowers them in battling this disease. This enables continuous monitoring of health, ultimately leading to improved health outcomes [29].

Community-Based Initiative

Community engagement plays a vital role by actively involving patients from the underserved community reduces the cancer disparities [30]. This focuses on involving individuals in their healthcare through a local support system. These initiatives create a supportive environment where patients feel empowered and motivated to take charge of their health. Health awareness campaigns, organizing local events, mobile health clinics, peer networking, support groups, and health camps, which result in patient education, health awareness, and help in early diagnosis and intervention. In India, cultural sensitivity and shyness often create a barrier to discussing topics like breast cancer. To raise awareness in a respectful yet impactful way, organizations like Tata Memorial Hospital introduced the “Gaanth Pe Dhyaan” campaign. The campaign cleverly links lumps in food, a familiar and non-threatening concept, with breast lumps, helping communities to understand the importance of early detection. By using relatable everyday experiences, the initiative breaks the taboo, encourages conversation, and promotes proactive health checkups without offending cultural sensibilities [31]. HCG Cancer Center launches the “Power of Good Wishes” initiative on World Cancer Day 2024 to inspire hope and positivity among cancer patients. As part of this program, each patient receives a box containing an empty bottle and 12 chits. Every month, they write down a wish and place it in the bottle. After a year, during the next World Cancer Day, they open the chits to reflect on their journey and progress.

Patient-Centered Communication and Relationship Building

Communication acts to connect healthcare providers and patients, fostering trust and helping in relationship building [32]. Patient-centered communication fosters the collaborative dynamics between physicians, patients, and families, ensuring that care decisions are guided by shared goals. This approach prioritizes understanding the patient's beliefs, concerns, and preferences while considering the broader bio-psycho-social factors influencing their health. establishing trust, demonstrating mutual respect, and conveying medical information in a clear and accessible manner [33]. Patient portals allow cancer patients to effectively communicate with their healthcare providers, leading to better health outcomes as the patient [34].

A key aspect of relationship building in healthcare is recognizing and respecting patients as individuals, valuing their unique perspectives, and acknowledging their unique preferences. Physicians who show genuine curiosity about their patients’ goals, needs, and emotions can ask more insightful questions, leading to a deeper understanding of their experiences. This, in turn, allows, more personalized, compassionate, and meaningful care. Additionally, providing undivided attention and actively listening to patients fosters an environment where they feel heard, valued, and supported, ultimately enhancing both communication and quality of care delivered [35].

Health Gamification and Incentive Programs

Gamification involves applying game-like elements in non-gaming settings, and in healthcare, known as health gamification, it serves multiple purposes. It can be used to train medical professionals and promote a healthier lifestyle among patients. By integrating features such as points, badges, a leaderboard, challenges, and rewards into health-related activities, gamification boosts engagement and motivation. This approach enhances patient education and encourages better health outcomes by making medical adherence and wellness efforts more interactive and rewarding [36]. In the Regional Cancer Center, Thiruvanathapuram, cancer patients receive a monthly pension of ₹ 1000 for lifelong supportive care after completing treatment, provided they belong to the below poverty line (BPL) category. To continue receiving the pension, they must obtain a certificate from their doctor [37].

Caregiver and Family Involvement

Cancer not only impacts patients but also affects the overall well-being of caregivers and families, particularly in areas such as communication about the illness, shifting family roles, and maintaining social support networks. Difficulties arise when patients and caregivers suppress their concerns and avoid discussing sensitive aspects of the disease and its treatment. Family caregivers often experience overload as they take on additional household and family responsibilities alongside their own [38].

Beyond emotional and social challenges, caregivers may also face physical health issues. While their health status may initially align with that of the general population, the demand of caregiving can lead to fatigue, sleep disturbances, and cognitive difficulties over time [39]. Caregivers often experience a deeper emotional impact than the patient themselves, as they provide both emotional and physical support throughout their treatment journey [40]. This underscores the importance of integrating supportive care services into oncology care. Psychosocial oncology, pain relief, integrative medicine, along with nutrition and rehabilitation, are vital for the well-being of both patients and their caregivers. These interventions not only help caregivers cope with emotional and physical strain but also reduce their burden, lower depression levels, and enhance their overall well-being, satisfaction, and caregiving skills [40]. Embracing the importance of caregiver or their families in the treatment journey of the patient can help in enhancing patient experience and compliance with the treatment plan [41].

Challenges

Various strategies discussed above can lead to better patient engagement, ensure timely treatment, and enhance overall well-being, but not everyone can engage with healthcare services in the same way. Several barriers make it difficult for patients to access care, understand medical guidance, or take charge of their health. These obstacles can create a gap in treatment and reduce the effectiveness of healthcare interventions. The challenges that a patient encounters frequently include the following:

Literacy Level and Diversity of Population

India’s vast linguistic and cultural diversity, along with varying levels of education, make standardized healthcare communication challenging. Cancer patients are required to navigate complex medical information, understand their diagnosis, and make informed decisions about their treatment. They must familiarize themselves with medical terminology, provide consent for procedures, attend appointments on time, and seek medical assistance appropriately [42]. Cancer treatment involves a variety of approaches, ranging from short-term to long-term interventions, often requiring a combination of therapies such as surgery, preventive screenings, chemotherapy, and radiotherapy. Effective cancer care depends on adequate health literacy (HL) and proper care coordination to ensure the best outcomes [43]. However, limited HL in cancer patients is linked to lower screening participation, delayed diagnoses at more advanced stages, poor symptom management, and reduced adherence to treatment plans. Addressing these gaps through improved health education and support systems can enhance patient engagement and overall cancer care effectiveness [44].

Lack of Awareness and Motivation

Motivation holds a vital role in the success of treatment. As a result of breakthrough medical innovations, the survival rate of cancer patients continues to improve. Nowadays, oncology interventions are provided outside the hospitals. Although patient and their families acknowledge the advantage, their active participation in the healthcare program remains essential. However, the prolonged duration of cancer treatment leads to a decrease in patient well-being and an increased risk of mental health disorders. Therefore, understanding how cancer patients sustain and strengthen their motivation is vital for improving their overall health and treatment results [45].

In low and middle-income countries, cancer patients often have a worse prognosis than those in high-income nations. The disparity is primarily due to factors like limited awareness, late diagnosis, and unequal access to affordable treatment. Limited awareness leads to late-stage presentation at healthcare facilities, as seen in data from four major centers in India, where most cancer patients seek medical help only in the advanced stage [46]. Additionally, myths surrounding cancer treatment are widespread, with many fearing that surgical intervention could cause the disease to spread. Some patients even believe that the treatment itself is as harmful as the illness. The side effects of cancer therapies, including hair loss, breast resection, and open healed wounds, further contribute to this reluctance, making timely diagnosis and treatment even more challenging [47].

Digital Divide and Technology Barrier

While digitization has significantly improved global health-enabling early and accurate cancer diagnosis and expanding access to specialized care-its benefits are not uniformly realized in low-resource public health settings like India. Digital health is especially significant for young adults, as they are more proficient in technology, whereas older adults are generally less inclined to use computers. Additionally, literacy disparities across Indian states impact digital adoption. Households without mobile phones face increasing pressure to acquire one to maintain healthcare access. Furthermore, disparities in digital infrastructure across regions hinder the successful implementation of advanced technologies, especially those requiring constant internet connectivity in rural areas [48].

Increasing Out-of-Pocket Expenditure

As cancer cases continue to increase and more advanced, costly treatment becomes common, non-medical expenses like food, transportation, and lodging place an additional financial burden on patients, especially due to the uneven geographical distribution of cancer treatment facilities [49]. Out-of-pocket expenses (OOPE) for cancer treatment differ based on factors such as study period, type of cancer, and specific cost components. For instance, in 2006-2007, the OOPE for all cancer types was ₹36,812 in India. In 2014, the cost per hospitalization episode was estimated at US$357. By 2017-2018, the average OOPE for cancer treatment had increased further, exceeding ₹2,895 for outpatient care and ₹52,393 for inpatient care, highlighting the growing financial strain on patients and their families [50]. In 2018-19, the average out-of-pocket (OOP) expenditure for hospitalization related to cancer treatment in India was estimated at ₹85,595 [51]. The cost of treatment was notably lower in public hospitals, averaging ₹38,859, whereas private hospitals had a significantly higher mean OOPE expenditure of ₹115,771 [52]. In 2023, the average direct out-of-pocket expense (OOPE) for an outpatient consultant was approximately ₹8,053 (US$101), while the cost for each hospitalization was around ₹39,085 (US$492). On an annual basis, the direct OOPE per cancer patient was estimated to be ₹331,177 (US$4,171) [52]. As a result, many cancer-affected households are forced to rely on borrowing or selling assets to cover these rising treatment expenses, further exacerbating their financial distress [53]. 

Insufficient Medical Workforce

The healthcare staff is an essential part of the healthcare system, which can only operate effectively when well-trained and skilled healthcare providers are available [54]. The ratio of registered doctors per patient is 1:834 in India, and according to WHO, the recommended ratio of doctors and patients for India is 1:1000 [55], leading to a significant imbalance in the healthcare system. Due to the high patient load, doctors often struggle to allocate sufficient time to patients. As a result, patients don’t receive the necessary attention and information.

Scarcity of studies on the engagement of cancer patients

The majority of research in India among cancer patients is in clinical trials [56]. India has around 4000 anthropologically distinct groups reflecting the country’s vast and diverse genetic pool. The population has a unique blend of genetic, cultural, linguistic, and dietary habits [57]. Hence, the impact of patient engagement strategies differs widely. Studies abroad in hospitals with fewer resources have also shown that patient engagement is possible with the involvement of policy makers and hospital managers; however, this needs to be done in the Indian setting, focusing on cancer patients and their outcomes [58].

Limitations of the Study

This study provides valuable insights into cancer patient engagement strategies; however, several limitations must be acknowledged. First, there is a scarcity of peer-reviewed literature specifically focusing on patient engagement in oncology, which led the authors to rely on alternative sources such as hospital websites, blog posts, news articles, and industry reports. These sources, while informative, may carry an inherent bias and lack academic rigor. Second, the study was limited to articles published in English from 2010 onward, potentially excluding relevant work published in other languages or before this period. Third, the potential for authors’ bias in interpreting findings cannot be ruled out. Consequently, not all relevant strategies, models, or best practices may have been captured, and the breadth of patient and family engagement in oncology is not exhaustively discussed.

Recommendations

Patient engagement has existed for a long time, but its importance is now more evident due to a shift toward patient-centered care. While tier-1 and tier-2 cities are now adopting engagement strategies, rural areas are still underserved. Since this is the era of influencers, collaborating with public figures who have survived cancer can be an effective strategy to promote a cancer awareness campaign and inclusivity, much like the successful polio eradication campaign in India. Influencers can transform medical information into relatable personal narratives, helping to break down stigma while emphasizing the value of early diagnosis and treatment. Extending their influence via social media, TV advertisements, and community programs can promote behavioral change, encourage screenings, and raise awareness about cancer prevention.

Setting up digital health kiosks in panchayat offices can greatly enhance healthcare access in rural areas. This kiosk can enable telemedicine consultations in multiple languages, ensuring that individuals can easily understand and navigate healthcare services. By breaking the language barrier and providing remote medical support, one can empower communities with convenient, reliable, and timely health solutions. 

Artificial intelligence (AI) systems can help in analyzing patient data to identify the likelihood of hospital admissions, which is called predictive analysis [59]. Health care providers can proactively identify patients at risk and deliver timely intervention to prevent complications, leading to better health outcomes. 

Financial support by micro health insurance can turn into a revolutionary step if it can be implemented systematically. Implementation of micro health insurance can be achieved through collaboration between fintech firms, insurance providers, and telecom companies. By offering low insurance plans (as affordable as ₹10 to ₹50 per month), adoption can be encouraged among the underserved population. Seamless Aadhaar-linked electronic know your customer (e-KYC) and simple one-time password (OTP) registration with unified payments interface (UPI)-based claim processing will simplify the process and help in faster reimbursement directly into mobile wallets, while automatic deduction from mobile wallets can ensure timely payments. This will make healthcare more accessible and financially sustainable. 

Use of innovative virtual reality technology can offer educational briefing, which will help patients better understand their treatment, symptoms, and effectively manage their stress. It can also help in the visualization of complex medical concepts and interventions.

## Conclusions

Well-being and wisdom fuel a society’s progress and prosperity. Cancer is not just a medical condition; it is a life-altering journey that affects patients and their families physically, emotionally, and financially. India's fight against cancer is an intricate and ongoing challenge and requires a requiring a multidimensional approach to patient engagement. India has significantly advanced its patient engagement strategies in recent years, with events like the COVID pandemic serving as a catalyst for rapid adoption and adaptation. While digital advancements, community initiatives, caregiver involvement, patient education, relationship-building, and gamification are promising strategies, the country's healthcare system still faces barriers such as inadequate infrastructure, poor health literacy, workforce shortages, and limited cancer research, which hinder the seamless implementation of patient engagement strategies.

India’s progress in patient engagement can be greatly amplified with AI-driven technology and the use of virtual reality, enabling early diagnosis and personalized treatment through precision medicine and timely interventions. This blend of innovation and patient-focused care improves medical outcomes and treatment adherence. Initiatives like Micro Health Insurance can promote financial accessibility that minimizes OOPE, while digital health kiosks and rural community engagement programs can further extend oncology services to rural populations, ensuring equitable cancer care across the nation. By integrating these, India can transform its oncology landscape and can become a more inclusive, accessible, and patient-centric ecosystem. The future of cancer care in India holds immense potential, empowering patients to take an active role in their treatment journey and ultimately increasing survival rates across the nation.
